# Split spawning increases robustness of coral larval supply and inter-reef connectivity

**DOI:** 10.1038/s41467-019-11367-7

**Published:** 2019-08-01

**Authors:** Karlo Hock, Christopher Doropoulos, Rebecca Gorton, Scott A. Condie, Peter J. Mumby

**Affiliations:** 1 Marine Spatial Ecology Lab, School of Biological Sciences, The University of Queensland, Brisbane, St Lucia 4067 Australia; 2 ARC Centre of Excellence for Coral Reef Studies, The University of Queensland, Brisbane, St Lucia 4067 Australia; 3 Oceans & Atmosphere, Commonwealth Scientific and Industrial Research Organisation, St Lucia, 4067 Australia; 4 Oceans & Atmosphere, Commonwealth Scientific and Industrial Research Organisation, Hobart, 7000 Australia

**Keywords:** Biooceanography, Coral reefs, Ecological modelling, Coral reefs

## Abstract

Many habitat-building corals undergo mass synchronous spawning events. Yet, despite the enormous amounts of larvae produced, larval dispersal from a single spawning event and the reliability of larval supply are highly dependent on vagaries of ocean currents. However, colonies from the same population will occasionally spawn over successive months. These split spawning events likely help to realign reproduction events to favourable environmental conditions. Here, we show that split spawning may benefit corals by increasing the reliability of larval supply. By modelling the dispersal of coral larvae across Australia’s Great Barrier Reef, we find that split spawning increased the diversity of sources and reliability of larval supply the reefs could receive, especially in regions with low and intrinsically variable connectivity. Such increased larval supply might help counteract the expected declines in reproductive success associated with split spawning events.

## Abstract (web summary)

Corals occasionally split their spawning over two consecutive months rather than utilising a single annual event. Here, the authors model coral larval dispersal to show that split spawning may increase the reliability of larval supply to reefs, with implications for recovery from disturbances.

## Introduction

Habitat degradation and fragmentation result from natural and anthropogenic disturbances, causing the loss of structure that supports entire ecosystems and the services they provide^1–3^. In marine environments, biogenic habitat providers — including seagrasses, kelps, and corals — host thousands of species that benefit economies through ecosystem function^4,5^. For coral reefs, in particular, recent climate-related disturbances have vastly reduced coral populations through mass coral bleaching^6^, compounded by local pressures including storms and pest outbreaks^7^. Once damaged, a key driver to coral recolonization and subsequent habitat recovery is the supply of larvae from remnant populations^8,9^.

Due to the bipartite life-history of most marine organisms, and corals in particular, understanding larval dispersal and metapopulation connectivity is a central component of reef management^10–12^. Heterogeneity in the responses of ecosystems to large scale disturbances allows remnant, neighbouring populations to supply disturbed reefs where self-seeding is no longer possible due to the loss of the local reproductive population^13,14^ (but see ref. ^15^). In the absence of large environmental fluctuations, coral mass spawning events are closely tied to a lunar cycle and feature synchronised release of gametes by colonies of many species, aided by cues from light, temperature, wind, and tidal fluctuations^16–20^. Most colonies spawn in a single synchronised mass spawning event per year or season. But in some years, spawning is split over two consecutive months at the same location^21–24^ during which a substantial proportion of colonies of the same species, as well as across multiple species, will spawn in one month or the other. Hence, larval supply can occur more than once per reproductive season during split spawning years.

Connectivity in marine systems is inherently stochastic, with interannual variation in both the oceanographic forces and local conditions creating transient links among populations^25–27^. While the numbers of larvae released during coral mass spawning events are enormous^28^, dispersal is highly reliant on ocean currents at the time of spawning^29^. This creates interannual stochasticity in larval supply among reefs, increasing the variability of supply and decreasing the reliability of inter-population connectivity. It has been proposed that the biological mechanism for split spawning is to realign the timing of coral reproduction with optimal environmental conditions^21,24^, and may be driven by a disconnect between lunar cues that trigger spawning and environmental cues that drive gametogenesis^24^. It is also considered feasible, albeit in the absence of evidence, that reproductive success could be impaired during a split spawning event because of lower synchrony that potentially reduces the likelihood of fertilisation success, premature release of gametes (but see ref. ^24^), or enhanced risk that part of the event misses optimal environmental conditions^23,24,30^. Yet split spawning events could also benefit corals by reducing the temporal uncertainty in larval supply by making supply more consistent over the years, as well as increasing the diversity of source locations that can supply larvae to a reef. In short, some reefs might experience enhanced recruitment owing to the multiple opportunities for supply provided by split spawning.

Split spawning events do occur on Australia’s Great Barrier Reef (GBR)^21^. The GBR is a complex system of ~3800 interconnected reefs that span more than 2300 kilometres in latitude. Here, we recreate seven years of spawning patterns across the GBR and assess the effects of split spawning events on the spatial and temporal variability, as well as robustness of larval supply to individual reefs. We use oceanographic models to compare the patterns of dispersal of coral larvae with split spawning against hypothetical connectivity patterns that would be obtained if only a single spawning occurred in a year. We find that split spawning events increase the reliability of larval supply by providing a greater number of external larval sources that supply reefs as well as more years during which inter-reef connectivity is likely to occur.

## Results

### Coral connectivity simulations

**Fig. 1:**
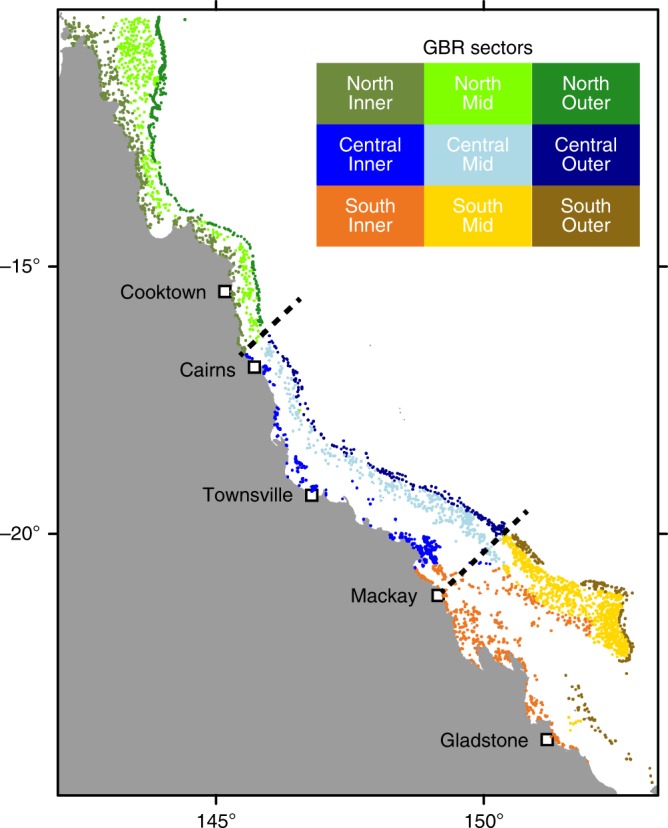
Dividing the Great Barrier Reef into geographical sectors. The colours of reef centroids show the division of reefs into northern, central and southern as well as inner-, mid-, and outer-shelf sectors. Thresholds used to simulate staggered latitudinal spawning events are shown as black dashed lines

We modelled coral connectivity on the GBR by using ~3800 polygons representing indicative reef boundaries^31^. Dispersal of coral larvae was driven by ocean circulation simulations^32,33^ with additional parameterisation based on coral larval development from laboratory studies^34^ and field observations^21^. To facilitate the interpretation of the results in terms of geographic regions as well as local conditions experienced by the reef^35^, we divided the reefs latitudinally into north, central and southern sections, and also according to their shelf position into inner-, mid-, and outer-shelf sectors (Fig. 1)^31^. Seven years of larval dispersal were simulated based on known timings of spawning throughout the reef and which included multiple split spawning events (Supplementary Table 1). During a split spawning year, corals would spawn in consecutive months within a given sector, which on the GBR means around 60% of colonies spawning in the first spawning month and 40% in the second^21^. We used four connectivity metrics to determine the effect of split spawning on potential larval supply: the relative levels of a reef’s external larval supply (i.e., supply coming from other reefs), the number of source reefs that supplied it, and the total number of years (out of a maximum of seven) as well as the period of consecutive years during which a reef was or was not supplied (i.e., the duration of failed external supply). Relative supply levels and the number of sources were calculated annually to capture both the mean expected value per reef and variability over time.

### Effects of split spawning on larval supply

**Fig. 2:**
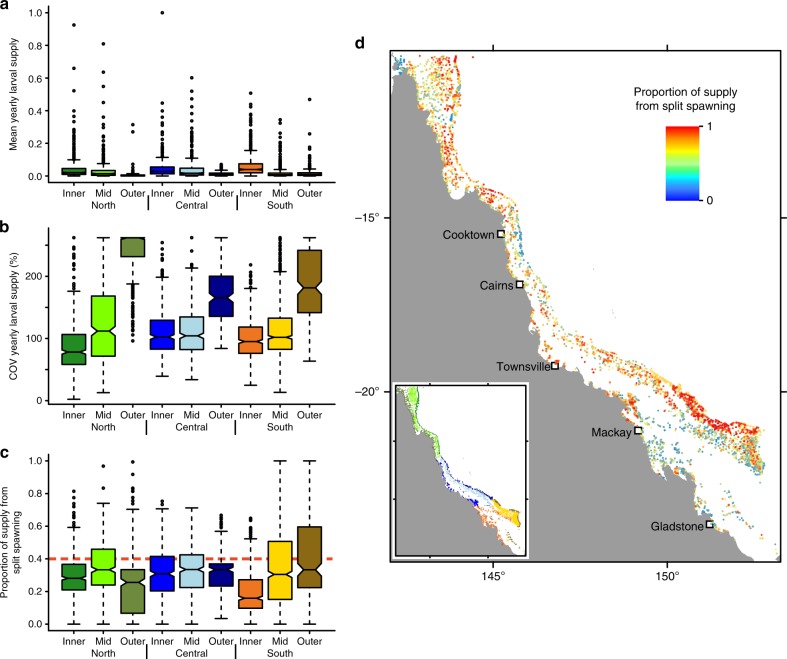
Patterns of larval supply and the effects of split spawning. Spatial patterns in (**a**) the relative magnitude of larval supply (normalised to 0–1), during the seven years of dispersal simulations, and (**b**) temporal variation in supply expressed as the coefficient of variation of relative supply per year. (**c**) Sensitivity of larval supply to split spawning as indicated by the mean proportion of annual coral larval supply contributed by the second spawning in split spawning scenarios during which 40% of larvae were released. Departures from the null expectation of 0.4 (red dashed line) imply that supply would have been highly sensitive to a spawning month had there been only a single spawning event. (**d**) Map showing locations of reefs and the mean proportion of supply contributed to each reef by the second spawning in the split spawning scenarios. The colours of the boxes in panels (**a**), (**b**) and (**c**) correspond to the colour scheme of the GBR sectors shown in the inset in panel (**d**). In boxplots, the centre line represents the median, the upper and lower limits of the box represent the third and first quartile respectively, the notches in the boxes represent the confidence interval around the median (equal to ±1.58 times interquartile range divided by square root of the sample size), the whiskers extend up to 1.5 times the interquartile range from the bounds of the box, and the points represent outliers beyond the limits of the whiskers. In (**d**) blue hues show reefs that received much of their larval supply in the first spawning of the year, red hues show reefs that received much of their larval supply in the second spawning of the year, and yellow hues show reefs that received comparable amounts of larval supply from both spawnings in a year. COV = coefficient of variation

Reefs had varying levels of mean annual larval supply (Fig. 2a). In general, inner- and mid-shelf reefs received more larvae than outer-shelf reefs. Potential for supply was most pronounced in the southern inner-shelf portion of the GBR where the shelf is widest and the potential sources are more numerous and/or closer. Supply was more variable over time in the outer-shelf regions throughout the GBR, as shown by a higher coefficient of variation (Fig. 2b).

If oceanographic conditions were similar between consecutive months of a split spawning event, we would expect the proportion of larvae arriving from the second month to equal that of the relative larval release (i.e., 0.4). Large departures from this proportion imply that the potential for larval supply differs dramatically between consecutive months. For example, if the proportion was 0.1 then 10% of all larvae supplied to the reef stem from the second month of spawning despite the fact that 40% of larvae were released in that month (and 90% of larvae stem from the first spawning month). Great disparity of proportional larval release vs. supply indicates that a single spawning event would have entailed elevated risk of failure. Continuing our hypothetical example, had a single spawning event occurred in the first month then larval supply would have been extraordinarily high because the proportion of supply exceeded release (0.9 vs. 0.6). But had corals spawned only in the second month then supply would have been weak because the reef received relatively few larvae in that month (0.1 vs. 0.4). Our results suggest that absolute levels of larval supply are not particularly sensitive to which month the corals spawn: the GBR-wide average proportion of supply in the second month was 0.36 (Fig. 2c), which is close to the null expectation of 0.4. There was, however, considerable geographic variation in the sensitivity of larval supply to the choice of month for spawning. Some reefs, in particular those in the inner-shelf regions of the southern GBR, received the bulk of their larval supply from the first spawning month in a year. In contrast, some outer-shelf reefs in the south received more larvae in the second month (Fig. 2d), suggesting that regional differences in oceanographic variability can affect the predictability of larval supply.

**Fig. 3:**
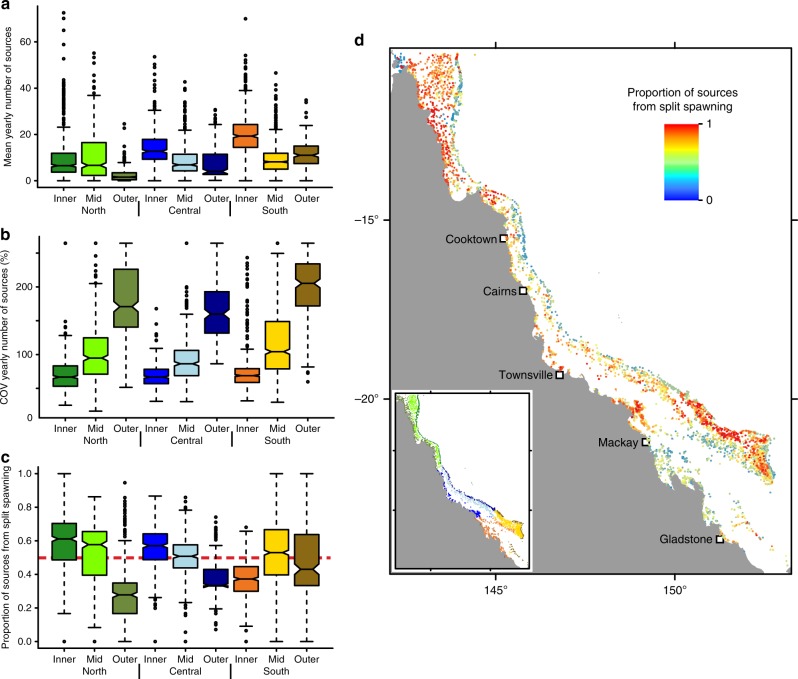
Patterns of the diversity of external larval sources and the effects of split spawning. Relative number of sources of coral larvae for reefs in each GBR section with split spawning summarized as (**a**) mean number of sources per year and (**b**) temporal variation in the number of sources expressed as the coefficient of variation of the annual number of sources. (**c**) Mean proportion of annual sources contributed by the second spawning in split spawning scenarios. For reference, a value of 0.5 (red dashed line) implies that an equal number — but a distinct set — of sources are provided by each consecutive spawning month. (**d**) Map showing locations of reefs and the mean proportion of sources reef contributed by the second spawning in the split spawning scenarios. The colours of the boxes in (**a**), (**b**) and (**c**) correspond to the colour scheme of the GBR sectors shown in the inset in (**d**). In boxplots, the centre line represents the median, the upper and lower limits of the box represent the third and first quartile respectively, the notches in the boxes represent the confidence interval around the median (equal to ±1.58 times interquartile range divided by square root of the sample size), the whiskers extend up to 1.5 times the interquartile range from the bounds of the box, and the points represent outliers beyond the limits of the whiskers. In (**d**) blue hues show reefs that had most of their larval sources in the first spawning of the year, red hues show reefs that had most of their larval sources in the second spawning of the year, and yellow hues show reefs that had comparable number of larval sources from both spawnings in a year. COV = coefficient of variation

Reefs in the outer-shelf regions tended to have fewer sources of coral larvae, particularly in the north (Fig. 3a). Supply from specific sources was also more variable among years in the outer-shelf regions throughout the entire GBR, as shown by the higher coefficient of variation in the number of source reefs per year (Fig. 3b). This pattern suggests that the number of sources to outer-shelf reefs in the north, although generally low, could vary considerably among years. Mid- and inner-shelf reefs exhibited much less variability in the annual number of sources. Unlike patterns of larval supply, split spawning had an important impact on the diversity of larval sources to a reef. On average, slightly over half (0.55) the sources were contributed by the second spawning month (Fig. 3c, d). Thus, most of the sources were unique to the either the first or the second spawning month, and split spawning essentially doubled the number of sources that would supply a reef in a year (Fig. 3c). However, in some regions, such as the northern outer-shelf and parts of the southern outer and mid-shelf, connectivity was considerably skewed towards one or the other spawning month (Fig. 3d).

**Fig. 4:**
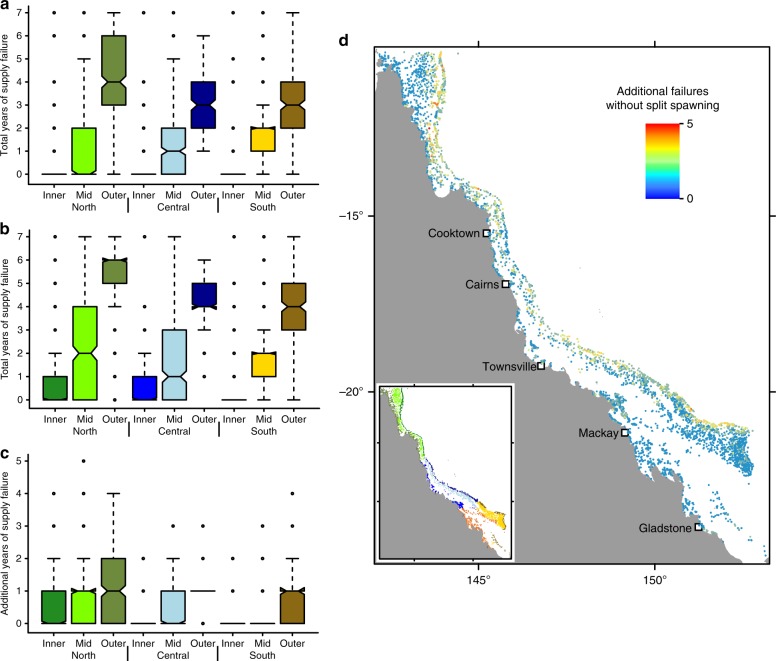
Effects of split spawning on reliability of larval supply from external sources. Number of years with external supply failure observed in the models (**a**) with, and (**b**) without, split spawning for reefs across sectors. Reefs with fewer supply failures would tend to have more reliable supply of coral larvae from external sources. (**c**) Additional years of supply failure the reefs would experience without split spawning. (**d**) Map showing locations of reefs that would experience more supply failure events if coral populations on the GBR did not undergo split spawning. The maximum number of years in all analyses was 7. The colours of the boxes in (**a**), (**b**), and (**c**) correspond to the colour scheme of the GBR sectors shown in the inset in (**d**). In boxplots, the centre line represents the median, the upper and lower limits of the box represent the third and first quartile respectively, the notches in the boxes represent the confidence interval around the median (equal to ±1.58 times interquartile range divided by square root of the sample size), the whiskers extend up to 1.5 times the interquartile range from the bounds of the box, and the points represent outliers beyond the limits of the whiskers. In (**d**) blue hues show reefs that would not have additional years of supply failure without split spawning, and red hues show the reefs that would have up to 5 additional years of supply failure without split spawning

Despite the majority of reefs being regularly supplied during the seven-year period of simulations, half of them (53%) experienced at least one year of failed larval supply from external sources even with split spawning. The vast majority of such failures occurred in mid- and outer-shelf regions (Fig. 4a, b). More than a third of reefs (36%) would experience at least one additional year of failed external supply in the absence of split spawning (Fig. 4c). There was again a marked cross-shelf trend, with inner-shelf reefs almost invariably having more reliable supply than outer-shelf reefs, and with mean number of years with supply failure being 3 or 4 for the latter even with split spawning. The importance of split spawning in preventing supply failure was greatest in mid- and outer-shelf regions (Fig. 4d).

**Fig. 5:**
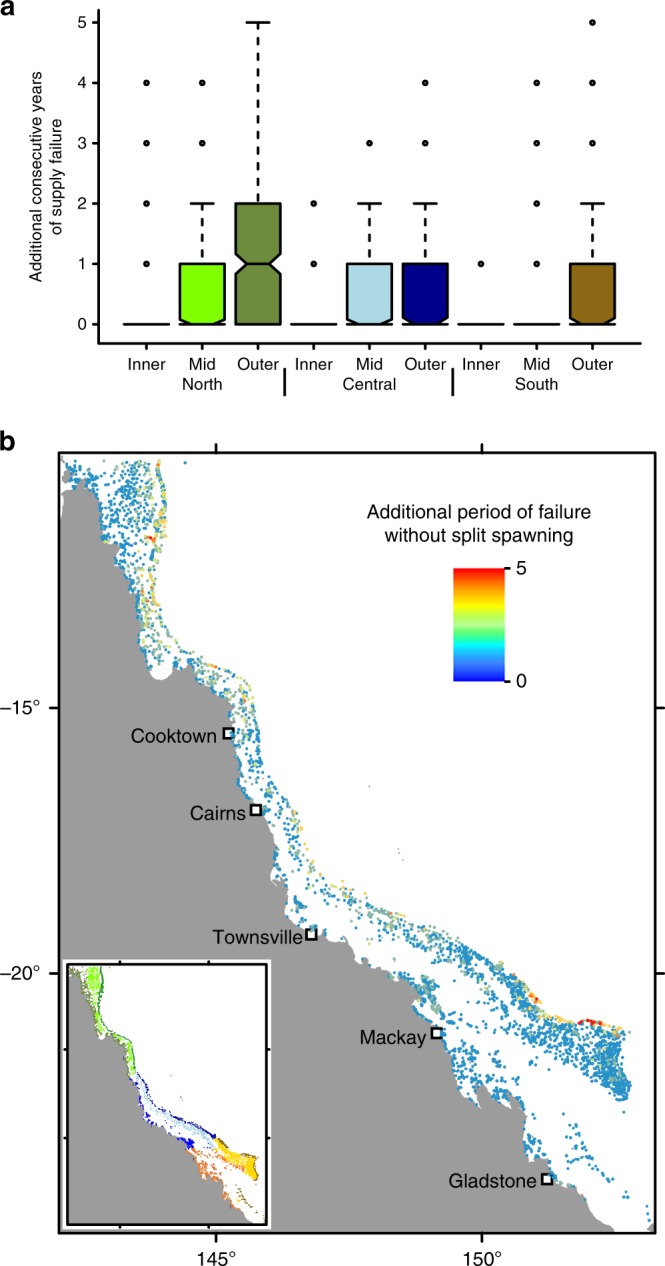
Effects of split spawning on the reliability of larval supply over time. Prolongation of the period during which supply fails without split spawning (**a**) by sector, and (**b**) as a continuous map. To guide interpretation, if a reef experienced two consecutive years of failed larval supply and split spawning prevented it from experiencing a third, then the ‘additional consecutive years of supply failure’ would be 1. The maximum number of consecutive years in all analyses was 7. The colours of the boxes in (**a**) correspond to the colour scheme of the GBR sectors shown in the inset in (**b**). In the boxplot, the centre line represents the median, the upper and lower limits of the box represent the third and first quartile respectively, the notches in the boxes represent the confidence interval around the median (equal to  ±1.58 times interquartile range divided by square root of the sample size), the whiskers extend up to 1.5 times the interquartile range from the bounds of the box, and the points represent outliers beyond the limits of the whiskers. In (**b**) blue hues show reefs that would not have a prolonged period of supply failure without split spawning, and red hues show the reefs that would have up to 5 additional consecutive years of supply failure without split spawning

Split spawning did not only prevent failures of larval supply in single years. Many reefs in outer- and mid-shelf regions would have experienced consecutive years of failure, which in rare cases could reach up to 5 years in the absence of split spawning (Fig. 5a, b). On average, split spawning prevents reduces the prolongation of failed larval supply by 0.4 years. Thus, split spawning may help reduce the period before recovery is initiated or supported by the external supply of larvae after disturbance impacts, especially on the outer shelf.

## Discussion

Coral connectivity on the GBR is highly variable, resulting in large fluctuations in potential larval supply between years. Patterns of connectivity exhibit strong cross-shelf trends with less consistent supply generally a characteristic of outer-shelf reefs. This stochasticity arises from variations in ocean currents and could lead to years with reduced external supply, especially for reefs in outer-shelf regions that appear to have fewer upstream sources. In general, the benefit of split spawning is to approximately double the number of larval sources to each reef and in so doing improve the reliability of larval supply. In the absence of split spawning, 36% of reefs would experience at least one year of failed external supply within seven years. Moreover, the average period of larval supply failure would extend by an average of 0.4 years in the absence of split spawning. Thus, split spawning ameliorates the inherent stochasticity of marine connectivity, and could play a role in enhancing the recovery potential of reefs in regions where connectivity tends to be low and/or highly variable, such as the outer-shelf regions of the northern and central GBR where the combination of oceanography and geography means that upstream sources of larvae are not readily available because currents tend to enter the reef matrix from the open ocean^36,37^. A more reliable supply over time from a wider pool of potential sources may be critical for recovery, especially in the early stages after a disturbance impact when populations are in greatest need of larval supply.

In some areas, strong deviation from the expected proportions of larval release versus subsequent supply indicates that these reefs would receive most of their larvae from only one of the two spawning events in a split spawning. Had corals utilised only a single spawning event the fate of larval supply would be relatively uncertain as the outcome would be highly dependent on the exact month when they spawned. Under these circumstances, split spawning effectively ‘hedges bets’, increasing the chance that at least one of the split events would coincide with favourable oceanographic conditions for larval dispersal and supply. Conversely, reefs that were close to the expected ratio of larval supply were more likely to be supplied equally during split spawning, and therefore had more consistent supply under various oceanographic conditions. Split spawning could still be beneficial to these reefs if, for example, a staggered supply increases the chance that recruits would survive density-dependent early post-settlement bottlenecks^38^. In particular, split spawning had a notably positive effect on the diversity of sources that supply a reef, as an additional spawning event within the same year has the potential to provide larvae from a substantially different set of source reefs. This increase in connectivity due to the second spawning event in the same year may increase the resilience of larval supply as more and more potential source reefs become degraded by disturbance impacts.

Mechanistically, split spawning has the benefit of realigning the timing of reproduction with optimal environmental conditions^21,24^. Yet, split spawning likely involves reproductive costs chiefly because of lower synchrony in gamete release among colonies, which elevates the risk of Allee effects with colonies potentially being too far apart to achieve high levels of successful fertilisation^39,40^. An intriguing possibility is that the benefits we describe here — that split spawning may improve the reliability of larval supply — might serve to offset some of the reproductive costs to fertilisation success during such events, as well as other aspects of lower larval concentrations such as reduced swamping of opportunistic predators^41^. It is too early to model such scenarios because of the absence of data, but continued insights into the biological causation of split spawning^17,24,42^ could enable formal testing of such ideas in future.

Although the outcomes of this study suggest that most of the GBR would enjoy some benefits in having more reliable supply from split spawning, the period examined was limited to the seven years of data available from the hydrodynamic models. A longer time series might refine these insights further; for example, the southern sector of the GBR only experienced three split spawning events over seven modelled years (see Supplementary Table 1). It is also possible that the reliability of supply to outer-shelf reefs may be more reliable than presented because this period included some strong El Niño years^43^ that contain ocean flow anomalies^44^. Constraining the model to just the GBR region may also underrepresent the amount of larvae some reefs would receive from reefs outside the GBR region, e.g., in the Torres Strait where potential sources could be close enough to provide demographically relevant supply. While these edge effects may mean that these reefs might normally receive more supply from external sources and be at a lower risk of supply failure than when considering the GBR alone, they may nevertheless also benefit from split spawning events in the same manner as other reefs.

The ecological importance of split spawning involves several major uncertainties. Split spawning may be affected by the warming seas^43^, as increased temperatures may interfere with conditions that determine the timing of spawning events. This could potentially lead to either more or less split spawning events, although observations in equatorial corals with less pronounced seasonality suggest that split spawning may remain common^42^. Finally, some species recruit less frequently than others and might exhibit storage effects^45,46^, which require rare external resupply only. How split spawning affects the demography of species that exhibit rare sexual recruitment is a consideration for future research.

Using connectivity models to infer the benefits of split spawning also comes with some important caveats. For example, just because a reef did not receive any external supply in the dispersal simulations does not mean that it experienced total recruitment failure in situ, since the resolution of the models does not allow for simulating fine-scale capture or fine-scale local retention of larvae. The models also make an assumption that the size of coral populations on source reefs can be inferred from the size of the reef, which may not reflect the actual extent of available coral habitat^47^. Moreover, the connectivity analyses did not include estimates of settlement and post-settlement processes, which are often key to quantifying realised recruitment^48^. These local conditions may overshadow what, from the connectivity models, appears to be a reliable supply of larvae to a reef, as would seem to be the case for many reefs in the inner-shelf regions where poor water quality imposes limits on survival of juvenile corals^49^. As such, space limitation during settlement or chronic mortality of settled corals resulting from poor local conditions may explain why inshore reefs remain in a relatively poor condition^49–53^ despite their apparently high potential for receiving external larval supply. In contrast, larval supply may be limiting on the outer shelf where the exposure to thermal stress and outbreaks of coral-eating crown-of-thorns starfish may be less pronounced^54^. This could also explain field observations of coral settlement on the GBR where the highest settlement has been recorded in the central GBR^55^.

In conclusion, we found that split spawning events could increase the robustness of larval supply to coral reefs. Such considerations may increase in importance as the GBR faces an increasingly uncertain future of escalating intensity and severity of disturbances^6,43,56–59^. Allee effects may become more frequent as densities of coral populations decline, exacerbating potential reproductive costs of split spawning. Under such circumstances, the benefits of split spawning to reliability of larval supply may play an increasingly important role in counteracting Allee effects. Moreover, altered oceanographic conditions due to climate change could have a major impact on future reliability of supply^60,61^, and split spawning may provide some resilience to coral populations against increasingly unpredictable hydrodynamic regimes. Therefore, in addition to helping coral colonies increase their reproductive success by aligning their reproduction with favourable conditions^21,24^, split spawning may also help coral populations on reefs better cope with inherent and potentially increasing stochasticity and fluctuations in oceanographic and disturbance regimes.

## Methods

### Spawning and dispersal simulations

Spatial displacement of the released particles was resolved using CONNIE, a high-resolution advection/diffusion model of the entire GBR region^32,62^. This model uses simulated ocean currents from the eReefs hydrodynamic model^33^ that are interpolated in time and space for each particle using a fourth-order Runge-Kutta scheme. The model used a three-dimensional structured mesh with a spatial resolution of 4 km for the forcing grid and a temporal resolution of one hour to simulate oceanographic currents, and also included the influence of the forces such as tides and wind. The fine-resolution simulation of the GBR and adjacent regions of the Coral Sea was embedded into a large-scale global circulation model to capture the influence of major ocean currents such as the South Equatorial Current and East Australian Current.

We simulated larval dispersal along the entire GBR during seven spawning seasons (GBR springs of 2008, 2010, 2011, 2012, 2014, 2015 and 2016) for which the oceanographic models are available (see http://www.csiro.au/connie/). *Acropora* corals were used as a model organism owing to its high abundance and importance as a reef builder and habitat provider^63^. *Acropora* are hermaphroditic spawners^23^ that release egg and sperm bundles in synchrony during mass spawning events into the water column for cross-fertilisation^16,41^. Recent meta-analysis has revealed that the major control on the timing of spawning is related to the rapid increase in seawater temperature^64^, and most corals spawn as waters are warming or close to the annual maxima^65^. Spawning generally occurs around the transition from spring to summer, peaking 4–6 days following the full moon, but local environmental factors influence timing^16^ and exact spawning times deviate around this general timing.

*Acropora* spawning times vary on the GBR along a latitudinal gradient, occurring earlier in the north and later in the south. Split spawning events also occur when the full moon is within the first half of the month^21^. The simulated coral spawning dates were therefore retrieved from the literature, field observations, and inferred from the full moon cycle when no direct observation could be gathered (Supplementary Table 1). To account for these differences in timing, we separated the release dates into three latitudinal sectors along the GBR — northern, central, and southern. The sectors have been designed to capture latitudinal differences in spawning times, mostly those observed by the various scientific stations on the GBR (Supplementary Table 1). Since the exact delineation of staggered latitudinal spawning is unknown, we defined 3 different latitudinal sector boundaries 125 km apart between northern-central and another 3 boundaries between central-southern sectors, yielding 9 different combinations of latitudinal sectors (Supplementary Fig. 1). Variation in connectivity outputs showed low levels of sensitivity to using different boundaries for latitudinal sectors (reef-wide connectivity metrics varied by 1–7% depending on the definition of latitudinal spawning sectors; Supplementary Table 2). The latitudinal sectors used in the analyses presented in the text are highlighted in Fig. 1 and Supplementary Fig. 1.

We simulated *Acropora* mass spawning by releasing 10^4^ larval particles from each reef over the course of the evening (20–23 h) on each of the predetermined spawning dates. The timing of spawning by latitudinal sector was implemented following the release dates in Supplementary Table 1. While *Acropora* colonies often spawn over a number of consecutive nights, to simplify the simulations we used a single spawning date at any combination of sector x season x month. This resulted in 25 discrete spawning dates (some occurring in more than one latitudinal sector) over the course of 7 reproductive seasons for which the oceanographic data are available. Even though the larval depth can change as a function of time due to changes in lipid content^66,67^, we presumed an upward swimming behaviour of developed larvae (i.e. coral planulae with cilia)^68^. Larvae were therefore driven by near-surface waters (upper metre of the water column) until they reached another reef. Larval competency initially occurs in *Acropora* ~4–5 days following spawning^34,67,69^, with peak competency generally occurring ~8–12 days after spawning^34,67^, followed by a prolonged exponential decline^34^. Competency and survival curves were incorporated to estimate the abundance of settlement on any given reef, derived using Equations 3 and 4 and Tables 1 and 2 from ref. ^34^ for *A. millepora*, a ubiquitous broadcast spawning coral on the GBR. Location of the particles was recorded in 12-hour intervals from the time of release for a period of 120 days. If at any time larvae were found to be located within a 1 km halo surrounding a reef^70^, they were considered to contribute to the larval pool of that reef. The amount of larvae a source reef contributed to a destination reef were used to estimate the strength of a directed connectivity link between source-destination pairs. The strength of links were also scaled to the size of source reefs, with the assumption that larger source reefs contribute more larvae and that this contribution is proportional to reef size. While linking the size of a spawning population to reef size does not take into account the possible state of the reef in terms of the relative abundance of live coral on it, this is a necessary assumption in the absence of a comprehensive dataset on coral populations that were, or may have been, present on each individual reef on any given year. The effect of local conditions at a destination reef on post-settlement mortality of *Acropora* recruits could not be reliably estimated GBR-wide and was therefore not modelled in the present set of simulations, instead assuming that the contribution to the post-settlement population was proportional to the received supply^54^. As a result, the link strength represented potential, rather than realised, connectivity among reefs.

### Estimating the effects of split spawning events

We used contributions of larvae from individual sources to obtain an estimate of larval supply that arrived at a destination reef as a result of a spawning event. Field observations of split spawning on the GBR indicate that ~60% of the colonies spawn during the first month, and the remaining 40% during the subsequent month^21^. To standardise the overall amount of larvae released per reef per year in the event of split spawning, we multiplied the strength of the outgoing links for reefs that underwent split spawning by the respective modifier (0.6 for the first spawning of the year and 0.4 for the second one). Finally, we combined the connectivity results for all spawning events during the same year in order to obtain GBR-wide annual connectivity networks.

To estimate the potential benefits of split spawning, we compared the relative contributions of the two yearly spawning events to the total connectivity for that year. For this, we assumed that all colonies on a reef would spawn in either the first or the second spawning month of a split spawning year. The difference in connectivity between the two scenarios was then used to determine how connectivity would have changed if a reef underwent only a single spawning event in a year. Supply from reefs in sectors that were not supposed to undergo split spawning in that year were assumed to be unchanged and were added to both values. We made no assumptions about whether an earlier or a later spawning event would have been a more likely outcome had split spawning not occurred. Instead, we examined how the value of connectivity metrics with either single spawning would deviate from the expected values recorded under a split spawning scenario.

### Connectivity network analysis

We used four connectivity metrics to determine the effect of split spawning on potential larval supply to the reef: (1) the relative amount and the coefficient of variation of coral larval supply a reef received from other reefs per year, (2) the number of individual reef sources that supplied it, (3) the total number of years during which it was/was not supplied, and (4) the longest sequence of successive years with no external larval supply over the course of the simulations. To compare larval supply across multiple years, we normalised the amount of supply a reef received per year by dividing it by the maximum annual supply received by any reef during that season, which resulted in values between 0 and 1 for each reef per year. While local retention was present in the simulations and a portion of the larvae would settle on the home reef, thus reducing the number of larvae available for dispersal to other reefs, it had little effect on the presented results given that all of the metrics rely on inter-reef connections (i.e., the number of reef-to-reef links and relative amount of larval transport among reefs), and not on self-connections. This kept the focus of the study on potential for reef-to-reef rescue via larval supply received from external sources. We then calculated per-reef means and coefficients of variation for supply to assess relative levels and temporal variation of annual external supply of coral larvae to reefs. We also compared the amount of external larval supply reefs received during individual spawnings in split spawning scenarios against a null expectation of 60:40 (i.e., the ratio of larvae released during the two yearly spawning events). Reefs with strong deviation from this null ratio were highly dependent on one or the other spawning event for the amount of larvae they received from other reefs in a given split spawning year irrespective of the actual ratio of released larvae.

We recorded the mean number of other reefs a reef could expect to have as external sources of larval supply per year, as well as coefficient of variation in the number of these external sources to estimate interannual variability. Since this metric was derived from an unweighted source count, the null expectation here was 50:50, i.e., that the two spawning events in split spawning will contribute the same number of sources to the yearly total. Reefs with strong deviation from this null ratio derived most of their connectivity from a single spawning in a split spawning year, while those that were close to the null expectation enjoyed increased diversity of sources by being supplied by two different sets of sources across the two spawning events in a split spawning year.

If a reef did not receive any external supply during a simulated spawning year, that year was counted as an external supply failure for that reef. Reefs with fewer years of external supply failure could, therefore, be considered to have a more reliable reef-to-reef supply of coral larvae even with high variation in interannual connectivity. We calculated the total number of years with external supply failure, as well as the number of successive failure years, for each reef. We also calculated the number of extra years without external supply, as well as the increase in the number of successive failure years a reef would experience without split spawning.

All connectivity outputs were divided into latitudinal as well as cross-shelf sectors (see Fig. 1) using the GBR bioregions outlined in the GBR zoning plan^31^ to better illustrate how split spawning impacts would vary with reef’s position in a seascape.

## Supplementary information

Supplementary Information

## Data Availability

Reef coordinates and other spatial information on the GBR reefs provided by the Great Barrier Reef Marine Park Authority, including topographic GIS layers, available at http://www.gbrmpa.gov.au/resources-and-publications/spatial-data-information-services. Hydrodynamic models developed by eReefs and provided by CSIRO; available at http://dapds00.nci.org.au/thredds/catalogs/fx3/catalog.html. Interactive visualisation of particle tracking simulations available via CSIRO’s Connie online interface at http://www.csiro.au/connie/. Additional outputs used in the analyses for this study are available at 10.5281/zenodo.2653244. Additional materials available from the authors upon reasonable request.

## Code availability

The code used for the analyses is available at 10.5281/zenodo.2653249.
