# Supplementary material for: Split spawning increases robustness of coral larval supply and inter-reef connectivity
Source: Nat Commun. 2019 Aug 1;10:3463. doi: 10.1038/s41467-019-11367-7 (PMC6671964; doi:10.1038/s41467-019-11367-7)
Supplement: Supplementary file 1 — Supplementary Information [file 41467_2019_11367_MOESM1_ESM.pdf]

Supplementary Information for:

Hock et al. “Split spawning increases robustness of coral larval supply and inter-reef connectivity”

Supplementary Tables

Supplementary Table 1. Timing of larvae releases in dispersal simulations designed to represent the mass coral spawning events on the GBR.

Year	Month	Day	DAFM	Sector	Reference
2008	November	18	5	N	Andrew Baird – pers. comm.
2008	November	18	5	C	[4]
2008	December	15	3	C	[5]
2008	December	15	3	S	Inferred
2010	October	28	5	N	Inferred
2010	October	28	5	C	[6 7]
2010	November	26	4	N	[8] ; Andrew Baird – pers. comm.
2010	November	29	8	S	[9]
2010	November	30	9	C	[6 10]
2011	October	17	5	N	Inferred
2011	November	15	5	N	[8,11]; Andrew Baird – pers. comm.
2011	November	16	6	C	[10]
2011	November	17	7	S	Matthew Nitschke – pers. comm.
2012	October	9	9	N	Inferred
2012	October	9	9	C	Inferred
2012	November	5	5	N	Andrew Baird – pers. comm.
2012	November	8	6	C	Inferred
2012	November	9	11	S	Eugenia Sampayo, Matthew Nitschke – pers. comm.
2012	December	5	7	S	Eugenia Sampayo – pers. comm.
2014	October	11	5	N	Inferred
2014	November	12	8	N	Andrew Baird - pers. comm.
2014	November	12	8	C	[12]
2014	November	14	10	S	Luis Gómez-Lemos - pers. comm.
2014	December	11	7	C	GBRMPA - Eye on the Reef - in situ; Gerard Ricardo – pers. comm.
2014	December	12	8	S	Inferred
2015	November	2	6	N	Inferred
2015	November	2	6	C	GBRMPA - Eye on the Reef - in situ
2015	November	5	5	S	Luis Gómez-Lemos - pers. comm.
2015	November	30	4	N	Rachel Woods - pers. comm.
2015	November	30	4	C	GBRMPA - Eye on the Reef - in situ
2015	November	30	4	S	Inferred
2016	November	17	3	N	Inferred
2016	November	17	3	C	GBRMPA - Eye on the Reef - in situ
2016	December	20	6	N	Rachel Woods - pers. comm.
2016	December	20	6	C	Inferred
2016	December	20	6	S	Inferred

Larvae releases in dispersal simulations were based on listed timings of *Acropora* spawning. Inferred release dates are based on the full moon cycle for the given region [1-3]. DAFM = days after full moon.

Supplementary Table 2. Sensitivity of connectivity metrics to the definition of the latitudinal spawning sector boundaries.

Metric	Minimum value for any definition of latitudinal sector	Maximum value for any definition of latitudinal sector	Percent difference (max/min)
Mean supply	0.0204	0.0206	1.2%
Mean number of sources	10.5597	11.2984	7%
Mean years of supply failure	1.4367	1.5126	5.3%
Mean consecutive years of supply failure	1.2383	1.3255	7.1%

The minimum and maximum values correspond to the GBR-wide means for the respective metrics for any of the 9 definitions of latitudinal spawning sectors. The percent difference shows the discrepancy between the minimum and maximum value.

Supplementary Figures

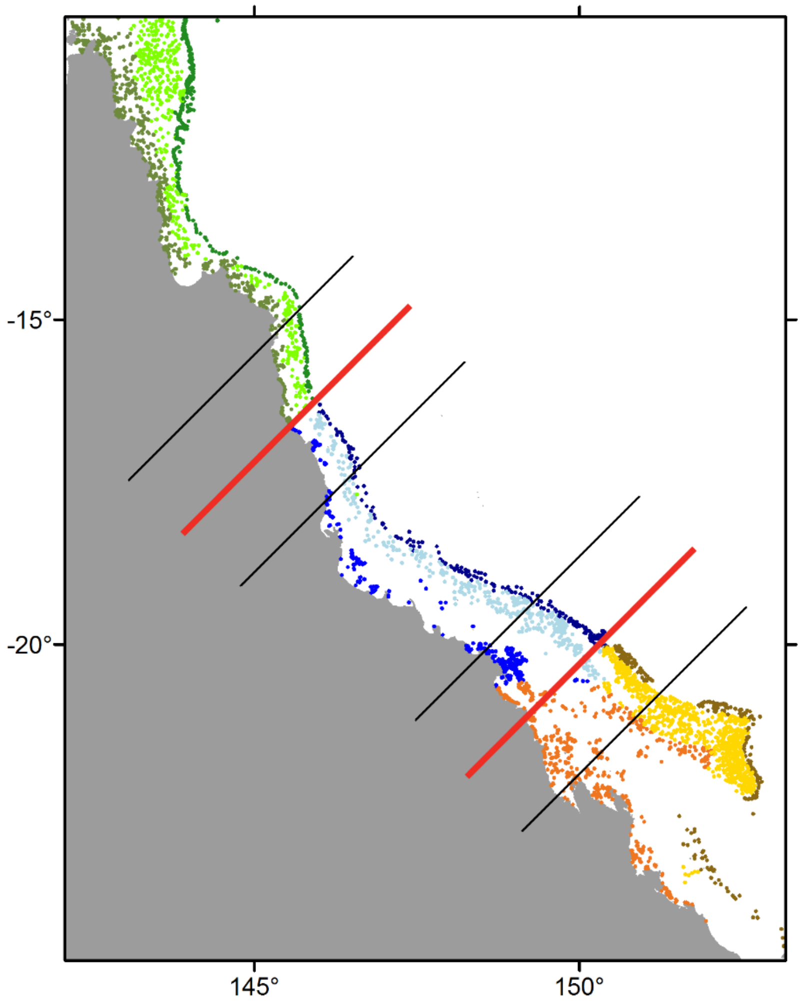

Supplementary Figure 1. Latitudinal sector boundaries tested in the sensitivity analysis. The distance between the upper and lower boundaries for a northern/southern set is 125km. The boundaries used in subsequent analysis and figures are shown as thick red lines.

References used in the Supplementary Material

1 Babcock, R. C. *et al.* Synchronous spawnings of 105 scleractinian coral species on the Great Barrier Reef. *Mar. Biol.* **90**, 379-394 (1986).
2 Harrison, P. L. *et al.* Mass spawning in tropical reef corals. *Science* **223**, 1186-1189 (1984).
3 Willis, B., Babcock, R., Harrison, P., Oliver, J. & Wallace, C. in *Proceedings of the 5th International Coral Reef Congress.* 343-348 (International Coral Reef Society, 1985).
4 Cumbo, V., Fan, T.-Y. & Edmunds, P. Scleractinian corals capture zooplankton within days of settlement and metamorphosis. *Coral Reefs* **31**, 1155-1155 (2012).
5 Graham, E. M., Baird, A. H., Connolly, S. R., Sewell, M. A. & Willis, B. L. Rapid declines in metabolism explain extended coral larval longevity. *Coral Reefs* **32**, 539-549 (2013).
6 Chua, C.-M., Leggat, W., Moya, A. & Baird, A. H. Near-future reductions in pH will have no consistent ecological effects on the early life-history stages of reef corals. *Mar. Ecol. Prog. Ser.* **486**, 143-151 (2013).
7 Palmer, C. V., Graham, E. M. & Baird, A. H. Immunity through early development of coral larvae. *Dev. Comp. Immunol.* **38**, 395-399 (2012).
8 Trapon, M. L., Pratchett, M. S., Hoey, A. S. & Baird, A. H. Influence of fish grazing and sedimentation on the early post-settlement survival of the tabular coral *Acropora cytherea*. *Coral Reefs* **32**, 1051-1059 (2013).
9 Doropoulos, C., Ward, S., Marshell, A., Diaz-Pulido, G. & Mumby, P. J. Interactions among chronic and acute impacts on coral recruits: the importance of size-escape thresholds. *Ecology* **93**, 2131-2138 (2012).
10 Siboni, N., Abrego, D., Motti, C. A., Tebben, J. & Harder, T. Gene expression patterns during the early stages of chemically induced larval metamorphosis and settlement of the coral Acropora millepora. *PLoS One* **9**, e91082 (2014).
11 Woolsey, E., Keith, S. A., Byrne, M., Schmidt-Roach, S. & Baird, A. Latitudinal variation in thermal tolerance thresholds of early life stages of corals. *Coral Reefs* **34**, 471-478 (2015).
12 Humanes, A., Noonan, S. H., Willis, B. L., Fabricius, K. E. & Negri, A. P. Cumulative effects of nutrient enrichment and elevated temperature compromise the early life history stages of the coral *Acropora tenuis*. *PloS ONE* **11**, e0161616 (2016).
